# Diagnosing the Information Limits of In Vitro Drug Release from PLGA Microparticle Data

**DOI:** 10.3390/pharmaceutics18070805

**Published:** 2026-06-29

**Authors:** Kushaan Sharma, Aryan Shah, Syna Sharma, Shreyan Shah, Mansoor A. Khan, Mariame Ali

**Affiliations:** 1 The University of Texas at Austin, 2515 Speedway, Austin, TX 78712, USA; 2 Texas A&M University, 400 Bizzell St., College Station, TX 77840, USA; aryanshah@tamu.edu; 3 University of Houston, 4302 University Dr., Houston, TX 77004, USA; synasharma10@gmail.com; 4 William P. Clements High School, 4200 Elkins Rd., Sugar Land, TX 77479, USA; shreytx@outlook.com; 5 Irma Lerma Rangel College of Pharmacy, Texas A&M University, 159 Reynolds Medical Building, Mail Stop 1114, College Station, TX 77843, USA; mkhan@tamu.edu; 6 Department of Pharmaceutical Sciences, School of Pharmacy, Texas A&M University, 206 Olsen Blvd., MS 1114, College Station, TX 77843, USA; mariame_ali@exchange.tamu.edu

**Keywords:** PLGA microparticles, drug release, burst release, machine learning, applicability domain, Korsmeyer–Peppas

## Abstract

**Background/Objectives**: Poly(lactic-co-glycolic acid) (PLGA) microparticles are widely used for sustained drug delivery, yet the release behavior reported in the literature remains difficult to predict across studies. It was hypothesized that this limitation reflects insufficient information content in commonly reported formulation variables rather than model inadequacy. **Methods**: A curated dataset of 321 PLGA microparticle formulations from 113 publications comprising 89 drugs and 4913 release observations was analyzed. Early time release was parameterized using Korsmeyer–Peppas descriptors (*n*, *K*), and burst release was quantified as the 24 h cumulative release. Machine learning models were evaluated using formulation-grouped cross-validation, applicability-domain analysis, and leave-one-study-out validation to assess cross-laboratory transportability. **Results**: Under formulation-grouped validation, predictability was limited (stacked ensemble: $R^{2}=0.156$ for *n*, $R^{2}=0.169$ for *K*, burst $R^{2}=0.100$). Leave-one-study-out validation yielded negative pooled $R^{2}$ values for all targets (−0.061, −0.040, and −0.180, respectively), indicating failure to generalize across laboratories. Applicability-domain filtering did not materially improve performance, supporting the interpretation that prediction is limited by missing or inconsistently reported variables rather than covariate extrapolation alone. **Conclusions**: These results reveal an information-limited regime in PLGA release prediction in which the literature covariates enable only weak formulation-level prediction under grouped validation and cannot support transferable models. Minimum reporting priorities are therefore proposed, including standardized characterization of polymer molecular weight, end-group chemistry, quantitative emulsification and solvent-removal parameters, and microstructural or porosity measurements, to enable reproducible formulation screening.

## 1. Introduction

Poly(lactic-co-glycolic acid) (PLGA) microparticles are among the most widely studied biodegradable carriers for sustained drug delivery and have become a foundational platform in controlled-release formulation research [1,2]. The appeal of PLGA systems (here, the polymer–drug microparticle formulation together with its release behavior) stems from their biodegradability and tunable release behavior, which can reduce dosing frequency and help maintain therapeutic exposure over extended periods [1,3]. PLGA was selected as the focus of this study because it is a particularly attractive matrix: it combines biodegradability with well-characterized biocompatibility and degradation kinetics that can be tuned through the lactide:glycolide ratio and polymer molecular weight, and it underpins many clinically established sustained-release and long-acting injectable products [1,4]. Among the range of controlled-release platforms (including liposomes, polymeric micelles, hydrogels, and solid implants), PLGA microparticles occupy a central position for injectable depot delivery of small molecules, peptides, and proteins because of this combination of tunability, degradability, and clinical precedent [1,2,5]. Despite this maturity, formulation development for PLGA microparticles remains heavily empirical because drug release is shaped by multiple coupled factors that vary across laboratories and manufacturing routes [3,6]. As a result, ostensibly similar PLGA systems can exhibit substantially different release profiles, complicating translation and slowing rational design [3].

Mechanistically, release from PLGA matrices reflects an interplay of drug diffusion, polymer hydration and degradation, pore formation, and erosion, with the relative contributions evolving over time [3]. Polymer composition (e.g., lactic:glycolic ratio), molecular weight, and end-group chemistry influence water uptake and hydrolysis kinetics, which, in turn, modulate microstructure development and release rates [1,3]. In parallel, micro- and meso-scale morphology, including particle size, porosity, and drug spatial distribution, strongly impacts early time release and the transition to degradation-controlled regimes [3]. Critically, many of these morphological attributes are not independent material properties: they are created during manufacturing. Common preparation methods can yield different internal structures depending on solvent choice, surfactant conditions, mixing and emulsification energy, and solvent removal dynamics; the most common routes are single-emulsion (oil-in-water, O/W) and double-emulsion (water-in-oil-in-water, W/O/W) solvent-evaporation or solvent-extraction methods, together with solid-in-oil-in-water (S/O/W) variants used for poorly water-soluble or solid drugs [6]. Consequently, process parameters can be important drivers of between-study variability, particularly when they are not reported in a standardized way [3,6].

A particularly important manifestation of this variability is *burst release*, the rapid early time release of a fraction of the drug, often associated with surface-localized payload and connected pore networks [7]. Burst behavior is frequently treated as a critical quality attribute in PLGA microparticle development because it can shape initial exposure and safety margins, even when long-term release remains acceptable [7,8]. However, burst is also highly sensitive to formulation microstructure and measurement conditions, making it challenging to compare across studies without careful standardization [3,7]. Even the in vitro release protocol can measurably influence the observed profile; for example, maintaining perfect sink conditions may alter PLGA degradation behavior and thereby affect release kinetics [9]. These experimental dependencies connect directly to broader challenges in establishing robust in vitro–in vivo relationships for complex non-oral drug products [10]. More broadly, rigorous in vitro release characterization remains central to formulation development across diverse delivery systems, from stimulus-responsive polymeric micelles [11] to in vitro assays combined with in vivo pharmacokinetic evaluation [12].

To connect this mechanistic complexity with formulation decisions, PLGA release is often summarized using mechanistic or semi-empirical models rather than by comparing every sampled time point directly. Mechanistic models, including reaction–diffusion formulations [13] and Monte Carlo erosion-based frameworks [14,15], represent the underlying physics explicitly and can explain individual systems in detail. However, each typically assumes a single dominant release mechanism and must be calibrated to system-specific parameters; when several coupled processes act together or shift over time, a model calibrated to one system transfers poorly to new formulations without refitting, so these models are powerful for explanation but limited for prediction [3]. Semi-empirical equations take the complementary approach: they deliberately compress complex release behavior into fitted descriptors that are easier to compare across formulations and laboratories. In this study, the Korsmeyer–Peppas power law was used in this role for the early time regime, summarizing each release curve by two fitted parameters (constants obtained by fitting the model to the data): the release exponent *n*, which describes the apparent early transport regime, and the rate constant *K*, which summarizes the release-rate scale within the fitted window [16,17]. Because PLGA release can involve overlapping diffusion, swelling, erosion, and degradation processes, these fitted values are best interpreted as compact early time curve descriptors rather than complete mechanisms for multiphasic release; they are therefore explanatory and comparable across studies but are not on their own predictive of how a new formulation will behave [3]. This gap is what motivates machine learning: while a fixed equation cannot learn the many interacting formulation and process effects and turn that complexity into prediction, a data-driven model can in principle do so, but only when the reported input data are consistent and complete enough to describe those effects. Accordingly, once heterogeneous release curves are reduced to comparable descriptors, ML can test whether routinely reported formulation covariates contain enough information to predict those descriptors across studies. Such targets can also support decision-making consistent with quality-by-design (QbD) frameworks, where the goal is not only prediction but also identification of controllable drivers and risk-relevant regimes [8].

In parallel, machine learning (ML) is increasingly used to accelerate formulation development by learning nonlinear relationships between composition, process, and performance from experimental data [18]. When datasets are information-rich and generated under standardized protocols, ML models have demonstrated utility for polymeric long-acting injectables and related controlled-release systems [19]. In this context, predictive accuracy itself is not the primary objective; rather, model behavior is used to interrogate the information content of reported formulation variables. Recent perspective work has also highlighted how ML could help navigate the design space and development bottlenecks for polymer microparticles in long-acting injectable contexts [5]. However, a key failure mode arises when models are trained on datasets derived from the published literature: the published record often omits or inconsistently reports critical process variables that determine microstructure (and therefore release), creating latent confounders that cap achievable performance [3,6]. In this setting, predictive performance may plateau at a finite level: models can capture reproducible structure, yet additional algorithmic complexity fails to substantially improve accuracy because the available covariates incompletely describe the microstructure-defining processes. Thus, limited performance does not imply that PLGA release is inherently unpredictable but rather that the published feature space may be insufficient to support fully quantitative prediction.

This motivates a shift in how ML is used on PLGA release data derived from the published literature. Rather than treating model performance as the final objective, ML can be used as an *instrument* to diagnose the limits of predictability imposed by reporting practices and study heterogeneity. A complementary concept from quantitative structure–activity relationship (QSAR) modeling is the *applicability domain* (AD), which characterizes where a model is expected to be reliable based on the similarity of new points to the training data [20,21]. In practice, leverage-based diagnostics (often visualized via Williams plots) are a common approach to flag potentially out-of-domain predictions and interpret residual behavior [22]. In heterogeneous datasets derived from the published literature, AD analysis can also be viewed as a map of the data manifold: it can test for subdomains where the literature is internally consistent enough to support stronger learning, even if global prediction remains limited [21,22].

In this work, a previously curated, literature-derived dataset of 321 PLGA microparticle release profiles was used to quantify the predictive ceiling attainable from commonly reported variables. Early time release was parameterized using Korsmeyer–Peppas descriptors [16,17] and 24 h burst behavior was quantified [7]; prediction was then evaluated under formulation-grouped validation to avoid within-formulation leakage (the unintended use of held-out information during training, which inflates apparent accuracy). Finally, applicability-domain analysis was applied to partition the dataset and assess the uniformity of model reliability across the domain [20,21,22]. By framing ML as a diagnostic tool for the information content of the published record, thus study provides concrete guidance on which experimental and process variables should be reported more consistently to improve predictability and to support risk-focused screening aligned with QbD principles [8].

## 2. Materials and Methods

### 2.1. Dataset Source and Composition

**Figure 1:**
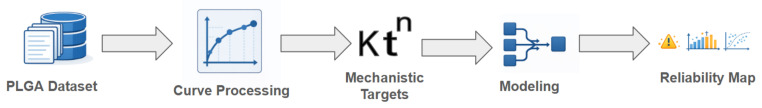
Study workflow for evaluating predictability limits in PLGA microparticle drug-release data. A published, multi-study dataset of in vitro release curves is reduced to physics-informed release descriptors (the Korsmeyer–Peppas parameters *n* and *K*, and the 24 h burst), which are then modeled under formulation-grouped validation and DOI-level leave-one-study-out validation. Reliability is assessed via generalization tests and diagnostics that quantify where prediction is stable versus fundamentally uncertain (e.g., applicability domain, study-level variance, and uncertainty proxies).

This study used a structured dataset of poly(lactic-*co*-glycolic acid) (PLGA) microparticle drug-release profiles and formulation descriptors derived from a prior systematic literature review (see Supplementary Table S1 for the full list of DOIs). In the original report, 1231 records were screened, and 113 studies were included, yielding 321 unique formulations across 89 drugs and 4913 release observations (5–48 time points per formulation; average ∼15). Each release observation corresponds to a single row indexed by a *Formulation Index* and a sampling time (Time, standardized to hours; Section 2.4), with an associated cumulative release value (Release, expressed as a fraction of cumulative drug released, 0–1). The overall analysis workflow is summarized in Figure 1.

The dataset focuses on stand-alone PLGA microparticles/microspheres with an in vitro cumulative release curve (minimum five reported time points), known drug identity (including SMILES for descriptor computation), and sufficient formulation metadata (e.g., polymer molecular weight, LA:GA ratio, particle size, loading/encapsulation). Studies were excluded when release was reported only in vivo, when systems were composite/hybrid dosage forms (e.g., hydrogels, scaffolds, implants) with confounded mechanisms, or when key formulation fields were not reported. The overarching goal was to evaluate how well machine learning models generalize across heterogeneous, multi-source literature data and to quantify the attainable predictive ceiling and its robustness to feature augmentation (the addition of further model input variables or features) [5,8,18,19]. Of the 321 curated formulations (compiled and quality-checked from the published literature), the paired Peppas modeling export (the subset of formulations yielding a valid fitted Korsmeyer–Peppas *n*–*K* pair) retained 300 formulation-level rows meeting joint target-quality and modeling-record criteria; 294 of these rows fell within the conventional 0≤n≤2 interpretability window and 6 had higher empirical fitted exponents. The retained numeric design matrices contained no missing feature values after preprocessing (here, filling in missing values and rescaling features). Burst_24h regression and classification used all 321 formulations.

### 2.2. Digitized Release Data Provenance and Uncertainty

The release profiles in the curated dataset were originally obtained from the primary literature as discrete (time, release) pairs. Most curves were digitized from published release-profile figures using WebPlotDigitizer (v4.8) [23], with a small minority extracted from tabular or supplementary numerical data when available. No explicit digitization-error propagation (e.g., repeated digitization trials or uncertainty bands) was reported; digitization/transcription uncertainty is therefore treated here as a component of the expected experimental heterogeneity in the literature-derived systems.

To reduce sensitivity to irregular sampling and to support consistent downstream target estimation, release curves were optionally resampled using monotone piecewise cubic Hermite interpolation (PCHIP), which preserves curve shape and monotonicity while generating uniformly sampled trajectories [24,25]. For mechanistic target estimation, *n* and *K* were computed from the original digitized points (after unit standardization and QC; Section 2.5) rather than from resampled trajectories to avoid interpolation-induced correlations. PCHIP resampling was used only for burst extraction at 24 h when a study did not report a measurement exactly at 24 h, and for visualization/standardization checks. Basic curve-level quality control excluded formulations with fewer than five sampled time points (insufficient resolution for stable kinetic estimation; Section 2.5). Fitted exponents above n=2 were treated as outside the conventional Korsmeyer–Peppas interpretability window, not as automatic deletion criteria for diagnostic modeling; the paired modeling export therefore retains these six high empirical fitted exponents directly. The observed prediction errors substantially exceed reported digitization uncertainty in comparable extraction studies [26], indicating that inter-study variability (manufacturing and reporting heterogeneity), rather than graphical extraction error, is the larger source of the effective noise floor in this heterogeneous literature compilation [2,3].

### 2.3. Unique Formulation and Formulation-Level Grouping

A *unique formulation* is defined as a single distinct value of Formulation Index, corresponding to one reported formulation instance (i.e., a unique combination of drug and key formulation attributes from a single source study). All time points sharing the same index are treated as a single release curve. To prevent temporal leakage (multiple time points from the same curve appearing in both training and test sets), formulation-grouped validation and holdout splitting used group-based strategies where Formulation Index is the grouping variable [27]. Thus, each formulation is confined to a single fold, ensuring that evaluation generalizes to entirely unseen formulations rather than interpolating over additional time points from known curves. In contrast, random splitting at the observation level would distribute time points from the same formulation across the training and test partitions, producing optimistic performance estimates through information leakage; formulation-grouped evaluation instead approximates the realistic deployment scenario of predicting release for formulations not seen during training [27].

For all predictive tasks, models were trained and evaluated on a formulation-level design matrix with exactly one row per Formulation Index (one prediction per formulation). The long-format (time, release) table was used only for target engineering and quality control (Section 2.5) and was not used as repeated training rows for formulation-level prediction to avoid implicit weighting of formulations with denser sampling.

### 2.4. Unit Conventions and Standardization

#### 2.4.1. Time Axis and Burst Definition

Release curves record Time as reported in the source study and Release as a fraction (0–1; slight overshoot above 1 can occur in reported cumulative data). All time values were converted to hours prior to burst extraction and kinetic fitting. When time was reported in minutes, days, or weeks, values were converted to hours using 1 min = 1/60 h, 1 day = 24 h, and 1 week = 168 h; studies with ambiguous time units were excluded. For burst analysis, Burst_24h is defined as the cumulative release at 24 h and is obtained by one-dimensional interpolation at the 24 h mark when a measurement at exactly 24 h is unavailable.

#### 2.4.2. Polymer Molecular Weight

Here, Polymer MW denotes the molar mass of the PLGA matrix polymer that constitutes the microparticle. This molar mass governs water uptake, chain mobility, and the rate of hydrolytic degradation, and hence release behavior. It is represented by a single numeric feature assembled from the literature-reported fields with the priority order $M_{w}$ (weight-average) >$M_{n}$ (number-average) > an unspecified molecular-weight field when neither $M_{w}$ nor $M_{n}$ is available. Molecular-weight values were converted to kDa when reported in Da. Values are otherwise used as reported; any residual heterogeneity in polymer molecular weight reporting is treated as part of the literature noise floor [1,3,6].

#### 2.4.3. LA:GA Ratio

The lactic-to-glycolic ratio is represented as a numeric feature (LA/GA) and used directly as LA_GA_numeric. Ratios reported textually in the source literature (e.g., “50:50”) were converted upstream into a single numeric ratio consistent with this representation.

### 2.5. Mechanistic Target Engineering

The release exponent *n*, the rate constant *K*, and the 24 h burst were selected as the primary predictive endpoints for three reasons. First, they compress heterogeneous, irregularly sampled release curves into a small set of mechanistically interpretable descriptors that are comparable across studies: *n* encodes the dominant early time transport regime (Fickian diffusion versus anomalous, erosion-coupled transport), and *K* summarizes the associated release rate [16,17]. Second, the 24 h burst is a widely used critical quality attribute for PLGA microparticles and is the most consistently reconstructable early-release scalar across studies with disparate sampling schedules [7]. Third, defining mechanistic targets rather than raw release percentages at arbitrary times avoids conflating differences in reporting time grids with differences in release behavior, which is essential when pooling multi-source literature data.

#### 2.5.1. Korsmeyer–Peppas Parameterization

Rather than learning release percentages at arbitrary times, the learning targets were defined as physics-informed parameters from the Korsmeyer–Peppas power-law model [16,17]:

(1) $$\frac{M_{t}}{M_{\infty }}=K\;t^{n},$$

where *n* is the release exponent (mechanistic regime indicator) and *K* is a rate constant. For each formulation, *n* and *K* were estimated using log–log linear regression of $\log(M_{t}/M_{\infty })$ versus log(t) over the sub-60% release region ($M_{t}/M_{\infty }\leq 0.60$) to reduce late-time saturation effects. Because the log–log fit is undefined at t=0 and at $M_{t}/M_{\infty }=0$, time points with t≤0 or $M_{t}/M_{\infty }\leq 0$ were excluded from the Peppas regression. When early time values were reported as exactly zero due to assay resolution, the first strictly positive release time point was used as the start of the fitting window. Fitted exponents above n=2 were flagged as outside the conventional interpretability window. This flag reflects the fact that the Korsmeyer–Peppas exponent retains a defined transport interpretation only across the Fickian-to-Case II range, so values far above this bound no longer correspond to a recognized physical transport regime and instead indicate that the single power law is being applied outside its early time validity. Such fits were nonetheless retained in the diagnostic paired modeling export when the fit otherwise met target-quality and modeling-record criteria. Formulations with fewer than five time points were excluded from mechanistic fitting to reduce unstable parameter estimates. Release profiles were not manually segmented into distinct kinetic phases; instead, restricting the fit to the early sub-60% window confined estimation to the diffusion-dominated regime in which the power law is theoretically valid, so later erosion- or degradation-controlled behavior in multiphasic or non-Fickian profiles was excluded by construction rather than explicitly modeled, with strongly anomalous fits (n>2) flagged as outside the interpretability window.

#### 2.5.2. Initial Burst Metric and Risk Classes

To address the known sensitivity of early time release to formulation and processing heterogeneity [2,3,7], Burst_24h was computed by interpolation at 24 h after time-unit standardization (Section 2.4). In addition to regression on Burst_24h, an operational burst-risk classification task binned formulations into two burst-risk regimes using a single threshold selected to separate lower/moderate early release from high early release:

- Class 0 (low/moderate burst): Burst_24h <20%.
- Class 1 (high burst): Burst_24h ≥20%.

A binary split, rather than a finer multi-class scheme, was adopted for two reasons. Operationally, the decision-relevant question in early formulation screening is whether a formulation exhibits acceptable versus excessive early release, for which a single low/moderate-versus-high boundary is the natural framing. Empirically, the compiled literature contained only five low-burst formulations, so a finer-grained scheme would only partition an already sparse minority into uninformative subgroups. The 20% value was used as a pragmatic operational threshold separating low/moderate from high early release. No class reweighting, resampling, or synthetic augmentation was applied; the classifier was trained directly on the available labeled formulations. Given the severe class imbalance observed in the compiled literature, classification results were interpreted using class-sensitive metrics rather than accuracy alone [28,29].

### 2.6. Feature Engineering and Model Matrix

A fixed, purely numeric feature set (p=15) was used for all learning tasks (mechanistic parameters and burst regression/classification). The feature set comprised Drug MW, Drug LogP, Drug TPSA, MolLogP, TPSA, ExactMolWt, NumHDonors, NumHAcceptors, RotatableBonds, Polymer MW, LA_GA_numeric, hydrophilicity index, particle size, drug loading capacity, and drug encapsulation efficiency. Chemical descriptors were computed from drug SMILES using RDKit [30]; overlapping mass descriptors were retained because they originate from distinct curated versus RDKit-derived representations and were treated as diagnostic covariates rather than mechanistic causal claims. Formulation and polymer descriptors were taken from curated literature fields. No automated feature selection, dimensionality reduction, or recursive feature elimination was applied; the 15-feature set was fixed a priori so that predictive performance reflects the information content of the commonly reported variables rather than the outcome of a data-driven feature search, and so that an identical design matrix supports the cross-validation, leave-one-study-out, applicability-domain, and uncertainty analyses.

In addition, a composite *hydrophilicity index* was defined as

(2) $$\;\mathrm{Hydrophilicity}\;\mathrm{Index}=\left(\frac{1}{\mathrm{LA}/\mathrm{GA}}\right)\left(\frac{1}{\mathrm{Polymer}\;\mathrm{MW}}\right),$$

to provide a monotonic proxy for polymer aqueous accessibility that captures the joint directionality of increased glycolide content (lower LA:GA) and reduced chain length on water penetration and degradation propensity [1,3]. This composite descriptor is used only as a directional monotonic encoding, not a physical quantity; it serves as a heuristic feature summarizing two well-established qualitative trends in PLGA behavior [1,3].

### 2.7. Formulation-Method Augmentation

To test whether predictive performance was limited by omission of high-level process information rather than intrinsic variability, a robustness analysis was performed by augmenting the feature set with a categorical Formulation Method variable. Detailed reported preparation routes were harmonized into canonical method categories where possible (e.g., O/W, S/O/W, W/O/W, and related emulsion-route labels) and encoded using one-hot encoding within the training folds. Harmonization yielded four canonical emulsion-route categories (O/W, S/O/W, W/O/W, and S/W/O/W) obtained by a deterministic mapping from the reported preparation descriptions; category definitions and formulation-level frequencies are provided in Supplementary Table S3. Because the analysis used a single pre-existing curated dataset and harmonization was performed as a deterministic category mapping rather than independent duplicate manual coding, formal inter-rater agreement could not be calculated; possible residual misclassification of these coarse method labels is therefore acknowledged as a limitation.

All preprocessing, encoding, and scaling were performed inside cross-validation folds to prevent information leakage. Model architectures, validation splits, and hyperparameters were unchanged from the primary analysis. The objective of this experiment was not to optimize performance but to test whether inclusion of a coarse process descriptor materially altered global predictability. Improvement limited to marginal changes in $R^{2}$ was interpreted as evidence that predictive performance is constrained by missing fine-grained structural descriptors rather than omission of high-level formulation categories.

### 2.8. Preprocessing, Model Families, and Specification

To avoid optimistic bias in a heterogeneous, literature-derived dataset, all preprocessing steps (imputation and scaling) were performed within-fold during validation. Every preprocessing transformation was fit on the training portion of each fold only [31]. Because missing or non-standard polymer molecular weights had already been resolved during dataset curation (Section 3.10), the curated 15-feature modeling matrix contained no missing numeric entries; the within-fold mean-imputation step (estimating and filling in any missing values) was therefore retained for pipeline generality but was effectively a no-op, whereas feature scaling was an active within-fold transformation. No missingness-indicator variables were included.

#### Model Families as Probes of Learnability

Regression targets (*n*, *K*, and Burst_24h) were modeled using a stacked ensemble (a model that combines several base models into one predictor) comprising three base learners, random forests, gradient-boosted trees, and an RBF-kernel support vector regressor, with a linear ridge meta-learner [32,33,34,35,36]. Hyperparameters were fixed (no grid search or Bayesian tuning) to emphasize interpretability and to quantify information limits under realistic inter-study heterogeneity rather than to optimize benchmark performance. The ensemble was selected to span tree-based, kernel-based, and linear hypothesis classes, allowing predictive consistency across model families to be interpreted as evidence of a learnable signal rather than model-specific fitting. The stacked ensemble was retained as the primary diagnostic model because it provides a single heterogeneous model family for CV, LOSO, AD, and uncertainty analyses; benchmark models are reported separately to show that the observed information ceiling is not an artifact of this choice.

To prevent stacking leakage, the meta-learner was trained only on out-of-fold predictions of the base learners generated within the training portion of each outer validation split. Specifically, within each outer grouped fold, base-learner predictions used to train the meta-learner were produced via an inner grouped cross-validation on the outer training set only; no samples from the outer test fold were used to fit the base learners or the meta-learner. Final predictions for the held-out outer fold were generated by refitting base learners on the full outer-training set and applying the trained meta-learner.

For benchmarking, baseline model families were also evaluated under the same grouped cross-validation protocol: ordinary least-squares linear regression, a random forest regressor, and gradient-boosted decision trees (XGBoost) [32,36]. These baselines were used diagnostically to assess whether predictive performance was consistent across structurally distinct hypothesis classes.

Burst-risk classes were predicted using a gradient-boosted tree classifier (XGBoost) with a binary logistic objective [36]. As above, hyperparameters were fixed by design to preserve the models’ role as analytical probes rather than optimization targets.

### 2.9. Validation Protocol

Model performance was assessed using grouped cross-validation, where the grouping variable was Formulation Index (Section 2.3). Regression performance was summarized using $R^{2}$, MAE, and RMSE computed from pooled out-of-fold predictions at the formulation level. A separate grouped holdout split (80/20 by formulation) was used as an additional leakage check for the burst-risk classifier. As a positive control on the validation machinery, a synthetic target was also constructed as a known linear function of the input features and processed through the identical grouped cross-validation and LOSO pipelines (including within-fold scaling and imputation); a correctly implemented workflow should recover this injected signal almost perfectly.

To test generalization across laboratories, leave-one-study-out (LOSO) validation was additionally performed at the DOI level. Each iteration held out one entire study, trained the stacked ensemble on the remaining studies, and evaluated on the held-out DOI. Regression performance under LOSO was reported using pooled $R^{2}$, MAE, and RMSE across all held-out studies.

For burst-risk classification, performance was summarized using the confusion matrix, per-class precision and recall, and macro-averaged F1 (macro-F1), computed from grouped predictions to avoid within-formulation leakage [28,29].

### 2.10. Variance Decomposition by Study Identity (ICC-1)

To quantify the proportion of variance attributable to study-level effects (proxies for laboratory-specific processing differences), a one-way random-effects analysis (ICC-1) was performed across studies that contributed at least 2 formulations. For each target (*n*, *K*, and Burst_24h), variance components were estimated using a one-way random-effects model, yielding $\sigma_{\mathrm{between}}^{2}$ and $\sigma_{\mathrm{within}}^{2}$. ICC(1) was computed as

(3) $$\mathrm{ICC}\left(1\right)=\frac{\sigma_{\mathrm{between}}^{2}}{\sigma_{\mathrm{between}}^{2}+\sigma_{\mathrm{within}}^{2}}$$

and inference followed standard ICC formulations for one-way random-effects designs [37,38]. An ANOVA *F*-test was used to assess whether between-study variability exceeded within-study variability, and results were reported for the subset of studies meeting the minimum formulation-count criterion.

### 2.11. Applicability Domain and Uncertainty Quantification

Given the risk of extrapolation in QSAR and QSPR style modeling on heterogeneous chemical space, applicability-domain (AD) analysis was used to separate “in-domain” predictions from high-leverage extrapolations [20,21,22,39,40]. Leverage values were computed from the standardized feature matrix *X* as the diagonal of the hat matrix $H=X{\left(X^{⊤}X\right)}^{-1}X^{⊤}$ (Williams-plot analysis). A warning leverage threshold was used to flag extrapolative points, defined as $h^{*}=3p/N$, where *p* is the number of predictor features and *N* is the number of unique formulations evaluated for a given target. Performance was reported separately for formulations inside and outside the warning leverage region to test whether the AD behaves as a monotone safety filter or instead reveals heterogeneous islands of predictability across literature sub-domains [21,22].

Prediction uncertainty was quantified using an ensemble-disagreement proxy: for each formulation, the uncertainty score was defined as the standard deviation of the base-learner predictions within the stacked model. This uncertainty was not calibrated to probabilistic coverage; it was used diagnostically to test whether error increases with model disagreement, consistent with the study’s emphasis on reliability over headline accuracy.

### 2.12. Reporting-Gap Analysis and Feature Importance

To relate predictive performance to reporting practices in the literature, variable availability across the compiled studies was quantified and compared with predictive importance and mechanistic relevance. Availability percentages were computed at the formulation level after harmonization of synonymous reporting fields. In particular, polymer molecular weight was represented as a composite feature using the priority order $M_{w}>M_{n}>$ unspecified molecular weight; if multiple molecular-weight fields appeared within a study, the harmonized value followed this priority rule. Mechanistic relevance was grounded in established PLGA release pathways and the known sensitivity of early release to microstructure and processing conditions [2,3,6,7,15].

Feature importance values for the prediction of the release exponent *n* were derived from a random forest regressor using standard impurity-based importance scores (mean decrease in impurity, a tree-model measure of how much each variable improves the model’s predictions; unrelated to chemical impurity) [32]. Importance values were computed on grouped cross-validation training folds and summarized as average percent contribution across features; only features exceeding a 5% importance threshold were reported.

Mechanistically relevant but unreported process variables (e.g., internal porosity, solvent removal rate, stirring/homogenization parameters, drying procedure, manufacturing scale, and polymer end-group chemistry) were identified from established PLGA release mechanisms and recorded as absent when not quantitatively specified in the source articles [2,3,6,7,15].

## 3. Results and Discussion

### 3.1. Dataset Overview and Feature Summary

The analyzed dataset comprises 321 unique PLGA microparticle formulations reported across 113 primary studies. In total, it contains 4913 release measurements spanning 89 different drugs, with numerical values originally extracted from graphical literature sources using digitization tools [23,26]. Individual formulations contributed between 5 and 48 sampling time points (average ∼15 per curve), indicating substantial variation in reporting density across studies. The observations were organized into a fixed-design matrix with 15 numerical covariates.

The composition of the curated dataset is summarized in Table 1. Emulsion-based oil-in-water (O/W) preparation dominated (63.6% of formulations), followed by solid-in-oil-in-water (S/O/W, 23.1%) and water-in-oil-in-water (W/O/W, 13.1%) routes. Polymer composition was concentrated at the two most common lactide:glycolide ratios, 50:50 (70.7%) and 75:25 (26.5%). Polymer molecular weight was most often reported as a weight-average ($M_{w}$, 59.2% of formulations), whereas number-average ($M_{n}$, 5.3%) and polydispersity index (4.0%) were rarely reported, and a substantial fraction (39.6%) was reported only as an unspecified molecular weight. The encapsulated drugs spanned a broad physicochemical range, with most molecules in the 300–600 Da range (66.4%) and moderately to highly lipophilic (LogP >0 for 88.2%). The digitized release-profile window was concentrated at early times, with 71.3% of formulations characterized over 14–56 h, consistent with this study’s focus on early time release and burst behavior.

To characterize the drugs, RDKit was used to derive physicochemical descriptors from SMILES strings [30]. These included molecular weight, lipophilicity (MolLogP), topological polar surface area, rotatable bonds, and hydrogen-bond counts. These were supplemented with additional drug properties as reported in the source papers. The remaining features describe formulation and polymer attributes commonly reported for PLGA microparticles, including polymer molecular weight and LA:GA ratio, particle size, drug loading and encapsulation efficiency, and a composite hydrophilicity index [1,2,3,6].

Korsmeyer–Peppas parameterization was successful for the majority of formulations after restricting the fit to the sub-60% release window, where this semi-empirical model is most interpretable during the early diffusion-dominated phase [16,17]. The paired Peppas modeling export retained 300 rows meeting joint target-quality and modeling-record criteria. Within this modeling subset, 294 rows had *n* values inside the conventional interpretability window (0≤n≤2), while six rows had higher empirical fitted exponents that are retained and examined in the predicted-versus-observed analysis below. Although *K* could be numerically estimated for 321 formulations before paired-modeling restriction, paired n/K modeling and the Peppas target summaries in Table 2 use the 300-formulation subset satisfying joint target-quality and modeling-matrix criteria; all predictive analyses of Peppas targets therefore use N=300.

The estimated modeling-target release exponent *n* had a mean of 0.71 (SD = 0.46). Larger *n* values in heterogeneous literature-derived data likely reflect combined diffusion/erosion behavior and fit sensitivity to protocol- and microstructure-level differences [3,9,17]. The modeling-target rate constant *K* averaged 0.21 (SD = 0.20). Burst_24h had a mean of 0.81 (SD = 0.20), indicating substantial early time release in most formulations, which is consistent with known surface- and porosity-driven burst mechanisms in PLGA microparticles [3,7,15] (Table 3).

### 3.2. Grouped Cross-Validation Performance of the Stacked Ensemble

All performance metrics were computed at the formulation level (one prediction per formulation). Grouped 10-fold cross-validation performance for the three regression targets is summarized in Table 4. To prevent within-formulation leakage, the *Formulation Index* was used as the grouping variable, which forces generalization to unseen formulation instances and is recommended when observations have clustered dependence structures [27]. The stacked ensemble uses model combination to improve robustness across heterogeneous regimes [35].

Both Peppas targets showed modest stacked-ensemble learnability, with *K* slightly higher by $R^{2}$ ($R^{2}=0.169$, MAE = 0.140, RMSE = 0.185; N=300) than the release exponent *n* ($R^{2}=0.156$, MAE = 0.306, RMSE = 0.420; N=300) (Table 4). Burst_24h exhibited weak recoverable signal ($R^{2}=0.100$, MAE = 0.144, RMSE = 0.186; N=321). These values support the interpretation that commonly reported covariates contain some signal but are insufficient for high-fidelity prediction in heterogeneous literature-derived PLGA data. For machine learning in pharmaceutical development more broadly, this implies that headline accuracies obtained from the literature-aggregated datasets can substantially overstate deployable performance unless they are validated under grouping that mirrors how models will be applied and that such models are better positioned as screening or hypothesis-generating tools than as quantitative predictors [5,18].

### 3.3. Benchmarking Against Baseline Model Families

**Figure 2:**
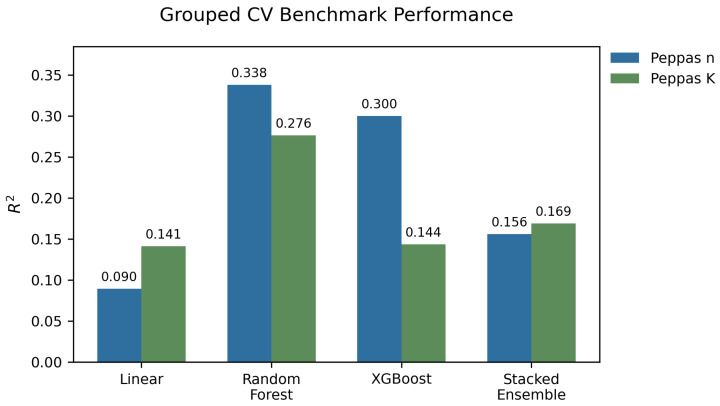
Benchmarking results for the release exponent *n* and rate constant *K* under grouped cross-validation.

The stacked ensemble was benchmarked against baseline models (linear regression, random forest, and gradient-boosted trees), using the same grouped cross-validation protocol (Table 5; Figure 2). Random forests and boosted trees are widely used as strong nonlinear baselines for tabular formulation data [32,36]. For the release exponent *n*, the random forest achieved the highest $R^{2}$ ($R^{2}=0.338$), followed by XGBoost ($R^{2}=0.300$), while the stacked ensemble achieved $R^{2}=0.156$. For the rate constant *K*, the random forest also performed best ($R^{2}=0.276$), compared with the stacked ensemble at $R^{2}=0.169$. For Burst_24h, the random forest achieved the highest grouped-CV $R^{2}$ ($R^{2}=0.188$), while the stacked ensemble reached $R^{2}=0.100$.

Despite these variations, performance remained modest across model families. This convergence is itself informative for this study, because the goal was to diagnose information limits in the literature rather than to optimize a single predictor. In this setting, a stable performance plateau across distinct hypothesis classes supports the interpretation that missing covariates and study-level shifts are the main error sources [5,18]. The stacked ensemble was therefore retained for the primary transportability and uncertainty analyses, while using the benchmark table to show that no model family removes the information ceiling.

This convergence is unlikely to reflect an implementation error. As a positive control, a synthetic target constructed as a known linear function of the same input features was recovered almost perfectly under both grouped 10-fold cross-validation ($R^{2}\approx 1.00$) and DOI-level LOSO ($R^{2}\approx 1.00$, evaluated on all N=321 formulations because the synthetic target is defined for every formulation), confirming that the folds, grouping, and within-fold scaling and imputation can recover a strong signal when one is present. The low observed $R^{2}$ values for the mechanistic and burst targets therefore reflect the limited information content of the reported covariates rather than fold mis-specification, scaling errors, or misapplied leakage controls.

### 3.4. Feature Importance

**Figure 3:**
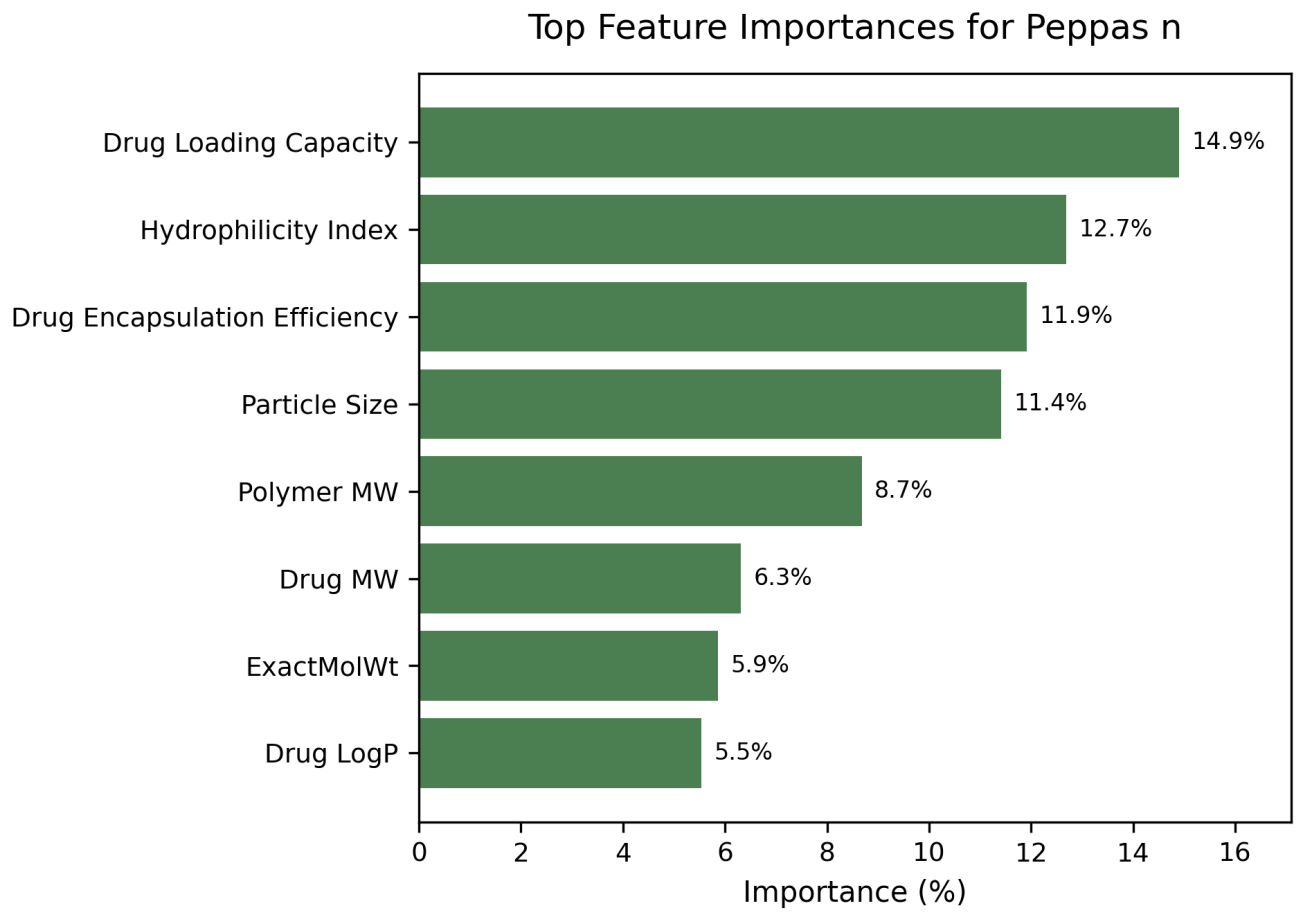
Top feature importance values for predicting the release exponent *n*.

Feature importance for the prediction of *n* is summarized in Table 6 and Figure 3. These percentages are normalized impurity-based importance values (mean decrease in impurity) from the random forest model, expressed as each feature’s share of the total importance (summing to 100%); they quantify each variable’s *predictive contribution* within the model rather than a causal effect size, and their purpose here is to identify which commonly reported variables carry the recoverable signal for *n*. Drug loading capacity accounted for the largest share (14.9%), followed by the hydrophilicity index (12.7%), drug encapsulation efficiency (11.9%), particle size (11.4%), and polymer molecular weight (8.7%). This ranking is mechanistically plausible: loading and encapsulation shape the initial drug distribution, particle size affects diffusion length scales, and polymer hydrophilicity and molecular weight influence water penetration and degradation kinetics [1,3,4]. By contrast, drug-level descriptors such as molecular weight and LogP contribute comparatively little to predicting *n*; the release exponent reflects the dominant early time transport *mechanism*, which is governed mainly by matrix microstructure and geometry (particle size, porosity, and polymer hydration) rather than by the diffusing molecule’s intrinsic properties, whereas drug physicochemistry more strongly modulates partitioning and the *magnitude* of release (and hence *K* or burst) than the shape of the transport regime captured by *n* [3,17]. The ranking also highlights a reporting asymmetry: the variables most likely to resolve release behavior are often reported only coarsely, while microstructure- and process-level drivers are largely absent. Notably, even though overall predictability is low, the recoverable importance is not diffuse but concentrated in a few variables: the five highest-ranked features account for roughly 60% of the total impurity-based importance, indicating that a small number of formulation and drug descriptors carry most of the learnable signal while the remainder contribute marginally.

To test whether additional methodological metadata could materially affect performance, one-hot-encoded harmonized formulation-method categories (e.g., O/W, S/O/W, W/O/W) were added as features. This yielded a negligible improvement ($\Delta R^{2}<0.01$), suggesting that method labels alone do not resolve microstructure-level variation unless accompanied by quantitative process descriptors (e.g., solvent removal kinetics, homogenization energy, and porosity measures) [6,7,15].

### 3.5. Predicted Versus Actual: Release Exponent n

**Figure 4:**
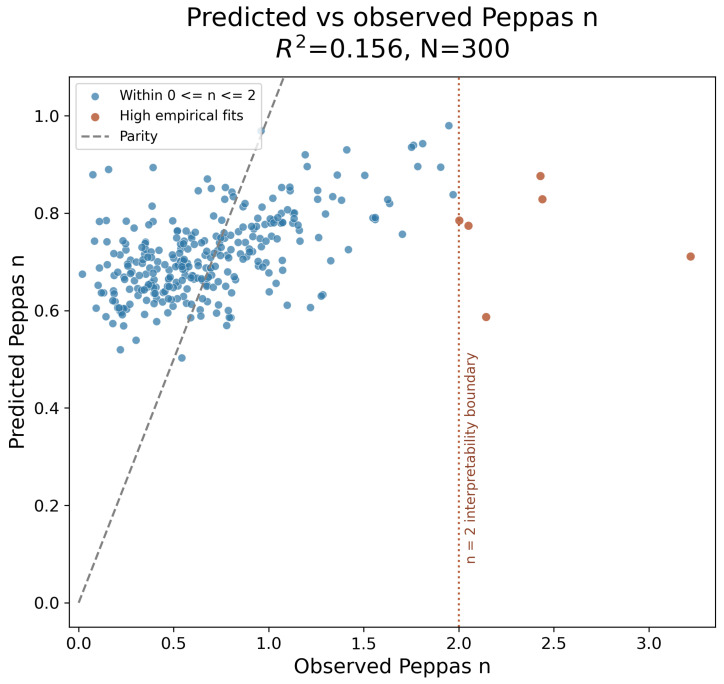
Predicted versus observed release exponent *n* (grouped cross-validation) for the paired Peppas modeling subset (N=300 formulations). The dashed diagonal indicates parity. High empirical fitted exponents visible above n=2 are retained in this diagnostic prediction export and are included in the Peppas *n* modeling-target summary in Table 2.

Predicted values for *n* captured only part of the observed variation and showed lower dispersion than the experimental observations (Figure 4). This contraction is consistent with regression-to-the-mean under heterogeneous measurement noise and latent study-level shifts, where the reported covariates capture only a subset of the mechanistic drivers [3,9]. Practically, the shrinkage indicates that the model is more reliable for ranking typical formulations than for confidently identifying extreme *n* regimes without additional microstructure or process descriptors.

Six formulations had fitted release exponents above the conventional n=2 ceiling (observed *n* from 2.00 to 3.22, spanning O/W and W/O/W routes and several drugs). These were retained as empirical, out-of-window fits and were not assigned a physical Korsmeyer–Peppas mechanism: exponents of this magnitude most plausibly reflect curve- or model-misspecification, multiphasic (diffusion-plus-erosion) release imperfectly captured by a single power law, or digitization noise in sparsely sampled early windows rather than a genuine super-Case II transport regime. A leave-the-six-out sensitivity analysis confirmed that these cases do not drive the central result: excluding them changed grouped-CV performance for *n* only modestly ($R^{2}$ from 0.156 to 0.191, MAE from 0.306 to 0.279, RMSE from 0.420 to 0.350), whereas the six cases evaluated alone were strongly mispredicted ($R^{2}=-15.5$), consistent with their interpretation as out-of-window empirical fits rather than a learnable mechanistic regime.

### 3.6. Burst Release: Regression and Classification

#### 3.6.1. Classification Results

**Figure 5:**
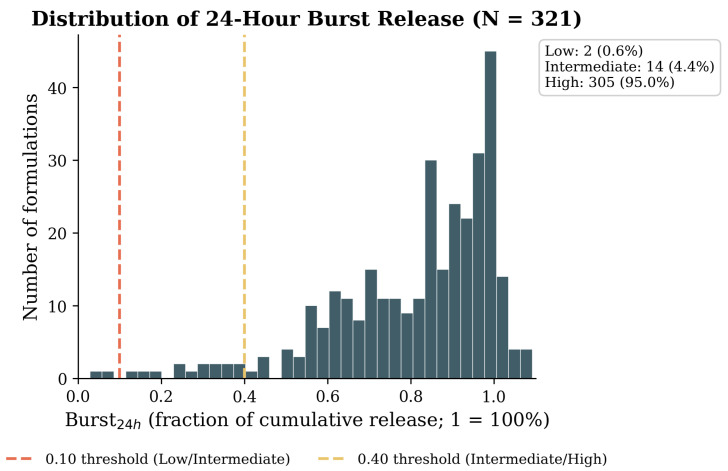
Distribution of 24 h burst release across the full dataset (N=321). The vast majority of formulations exhibit high burst under the binary 20% threshold, creating a class imbalance that limits discriminative modeling.

The 24 h burst release was the least predictable regression target ($R^{2}=0.100$). As a complementary diagnostic, a two-class burst-risk classification task was evaluated, with bins defined as low/moderate burst (<20%) and high burst (≥20%). At the formulation level (321 unique formulations), the distribution was highly skewed (Figure 5): 316 formulations (98.4%) were high-burst and 5 (1.6%) were low/moderate-burst. The confusion matrix (Table 7) confirmed that the model predicted only the majority class across all folds, yielding high apparent accuracy (0.984) but a macro-F1 of only 0.496. Macro-averaged metrics are appropriate in this setting because they penalize trivial majority-class predictors and reflect minority-class failure [28,29].

#### 3.6.2. Diagnostic Interpretation

This distribution is itself a diagnostic finding about the published record, not a model failure. The literature as compiled reflects typical PLGA microparticle outcomes under common emulsion-solvent evaporation conditions, where substantial early time release is often observed [3,6,7]. In this setting, classification provides a compact summary of dataset imbalance and is not intended as a deployable predictor of low-burst formulations.

Low-burst formulations are known to depend on microstructure- and process-level variables such as internal porosity, surface drug enrichment, solvent removal kinetics, and postprocessing steps, which are rarely quantified in sufficient detail to support cross-study prediction [3,7,15]. Consequently, the extreme imbalance renders standard classification uninformative. Standard remediation strategies such as synthetic oversampling (SMOTE), cost-sensitive loss weighting, or ordinal regression were considered [28,41]. They were deliberately not applied, since synthetic minority generation would create decision boundaries in sparsely populated regions of the feature space without adding a mechanistic signal. The degeneracy is therefore diagnostic: it reflects both the scarcity of low-burst formulations in the published record and the absence of quantitative process covariates needed to distinguish low-burst from high-burst outcomes (Section 3.10, Table 11).

### 3.7. Applicability Domain Analysis

**Figure 6:**
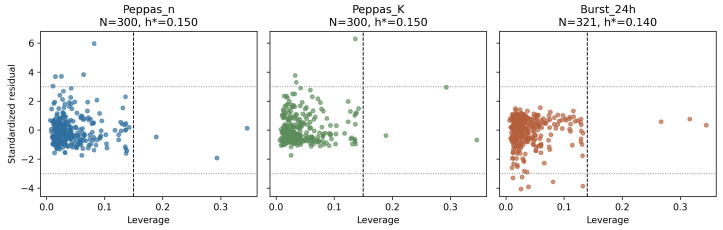
Williams plot for applicability-domain analysis (N=300 for Peppas targets, N=321 for Burst_24h). The vertical boundary corresponds to the warning leverage threshold $h^{*}$ computed for each target.

Applicability-domain (AD) analysis was performed using leverage-based Williams plots (Figure 6), with warning leverage threshold $h^{*}=3p/N$, where p=15 is the number of predictor features and *N* is the number of unique formulations evaluated for each target [20,21,22]. For the Peppas targets (N=300), $h^{*}=45/300=0.150$. For Burst_24h (N=321), $h^{*}=45/321\approx 0.140$. Table 8 reports the results.

**Figure 7:**
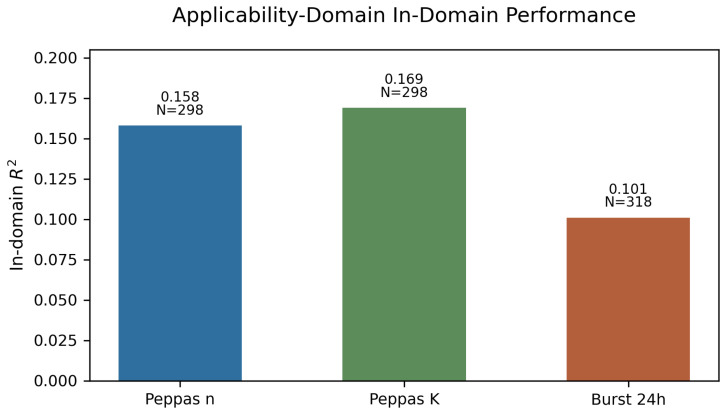
Applicability-domain analysis: formulation-level $R^{2}$ for in-domain formulations. High-leverage formulations are rare (N=3 per target), preventing distinct performance estimation for that subgroup.

For Peppas *n* and *K*, 297 of 300 formulations fall below the warning leverage threshold (in-domain), with 3 high-leverage outliers (Figure 7). For Burst_24h, 318 of 321 formulations are in-domain, with 3 high-leverage outliers. High-leverage formulations are therefore rare, indicating that most formulations lie within a chemically consistent covariate space as represented by reported features. The persistence of limited predictive performance within this in-domain region supports the interpretation that the performance gaps reflect unmeasured process and microstructure variables and study-level shifts rather than covariate extrapolation. This interpretation is consistent with best-practice guidance that emphasizes the AD as necessary but not sufficient for transportability when important causal drivers are unmeasured [20,39].

Two additional checks indicate that this conclusion is not an artifact of the chosen leverage threshold. First, tightening the warning threshold from 3p/N to 2p/N flags more formulations as high-leverage (e.g., 23 of 300 for the Peppas targets, versus 3 at 3p/N) but leaves in-domain performance essentially unchanged (Peppas *n*: $R^{2}=0.160$, MAE =0.305), with flagged points showing only marginally higher error (MAE =0.315). Second, leverage in the reported-covariate space is at best weakly associated with prediction error: the correlation between formulation leverage and absolute error is small for all targets (Pearson r=0.08 for *n*, 0.18 for *K*, and ≈0 for Burst_24h). A response-based check reinforces this decoupling: the worst-predicted decile of formulations by absolute error is markedly less accurate (e.g., MAE =0.95 for *n*) yet is not concentrated among the high-leverage points. Together, these results indicate that prediction failures are not explained by extrapolation in the measured covariate space, reinforcing the interpretation that the residual error likely reflects unmeasured process and study-level variables. A Mahalanobis-distance applicability domain (chi-square 95% cutoff) was additionally computed, which flagged 32 of 300 Peppas formulations and 35 of 321 Burst_24h formulations as out-of-domain. Mahalanobis distance was, however, only weakly associated with absolute error (Pearson r=0.05, −0.01, and −0.03 for *n*, *K*, and Burst_24h, respectively; all p>0.3), and out-of-domain error was not consistently higher than in-domain error across targets (for *n*, MAE =0.37 out-of-domain versus 0.30 in-domain, but the reverse for *K*). This mirrors the leverage-based result: distance-based domain definitions do not cleanly separate reliable from unreliable predictions in this dataset.

### 3.8. Leave-One-Study-Out Validation

To assess whether grouped cross-validation overestimates practical generalization, leave-one-study-out (LOSO) validation was performed. In this protocol, each of the 113 studies (unique DOIs) was held out in turn, the stacked ensemble was trained on the remaining studies, and evaluation was performed on the held-out study’s formulations. A cross-DOI duplicate check identified only two formulation pairs sharing identical features across different DOIs, indicating minimal direct feature-duplicate leakage.

**Figure 8:**
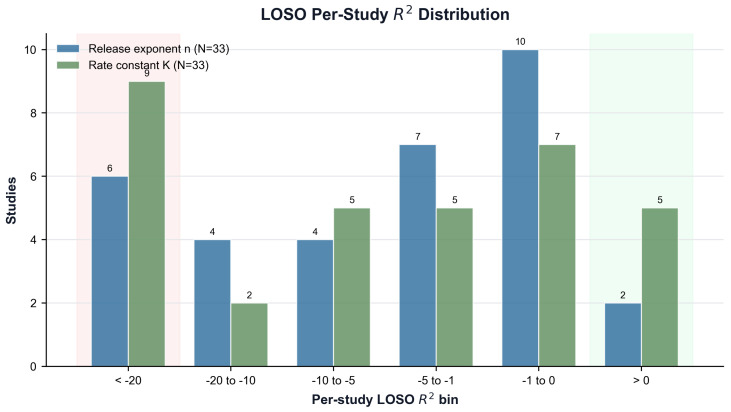
Binned distribution of per-study $R^{2}$ under LOSO validation for the release exponent *n* and rate constant *K*. Only studies contributing ≥3 formulations to the held-out set are included (33 studies for each Peppas target). Binning avoids compression by extreme negative per-study $R^{2}$ values while preserving the distribution of cross-study prediction failures. Pooled LOSO $R^{2}$ values were −0.061 for *n* and −0.040 for *K*.

LOSO produced substantially lower pooled $R^{2}$ than grouped 10-fold CV across all three targets (Table 9), with the binned per-study $R^{2}$ distribution shown in Figure 8. Error metrics worsened for *n* and Burst_24h, while *K* showed a negative LOSO $R^{2}$ despite similar absolute-error magnitudes, indicating degraded out-of-study explanatory power rather than a large increase in absolute error. For the release exponent *n*, LOSO $R^{2}=-0.061$ (MAE = 0.363, RMSE = 0.498), compared to grouped CV $R^{2}=0.156$ (MAE = 0.306, RMSE = 0.420). For *K*, LOSO $R^{2}=-0.040$ (MAE = 0.142, RMSE = 0.179), and for Burst_24h, LOSO $R^{2}=-0.180$ (MAE = 0.202, RMSE = 0.245). Negative pooled $R^{2}$ under LOSO indicates systematic study-level shifts, where models trained on reported covariates do not transfer reliably to unseen studies and can perform worse than a mean predictor out-of-study [39,40]. The contrast between formulation-grouped CV and LOSO localizes the performance collapse to the between-study variance component, consistent with heterogeneous in vitro conditions (e.g., sink conditions, medium changes, agitation, assay calibration) and unreported manufacturing details that influence microstructure and degradation [3,6,9].

The results are insensitive to the choice of imputation method used inside the cross-validation folds. In the raw literature records, a direct polymer molecular weight was missing or non-standard for some formulations; these values were filled once, during dataset curation and harmonization, before any model was fitted, so the final 15-feature processed modeling matrix was complete, with no missing values in any formulation whether used for training or testing. The fold-level imputation step therefore had nothing left to fill: re-running the full grouped 10-fold cross-validation and leave-one-study-out pipeline under median, *k*-nearest-neighbor, and mean-plus-missing-indicator imputation (in place of the default mean imputation) produced identical feature matrices and reproduced the grouped-CV and LOSO $R^{2}$ exactly for all three targets (for *n*, 0.156 and −0.061; for *K*, 0.169 and −0.040; for Burst_24h, 0.100 and −0.180). The negative cross-study performance is thus not an artifact of the imputation choice. To probe imputation-uncertainty propagation directly, a ridge-regression diagnostic was additionally run in which the curated polymer molecular-weight entries were deliberately re-masked and re-imputed under mean, *k*-nearest-neighbor, missing-indicator, and multiple imputation (20 draws). Across all four schemes, the leave-one-study-out $R^{2}$ varied within a narrow band and remained weak or negative for every target (Peppas *n*, −0.02 to 0.03; Peppas *K*, −0.04 to 0.07; Burst_24h, −0.30 to −0.20; multiple-imputation standard deviation below 0.025 $R^{2}$ units). Imputation uncertainty therefore introduces only minor variation and does not rescue cross-study generalization. This stress test used ridge regression as a fast diagnostic rather than the primary stacked ensemble and is reported as a sensitivity check; the primary leave-one-study-out values are unchanged.

The gap between LOSO and grouped CV quantifies the magnitude of study-level confounding. This gap is consistent with latent process variables (solvent removal rate, homogenization energy, internal porosity) and laboratory-specific implementation details shaping the learnable signal, although the present analyses establish association rather than proof of a specific causal driver (see the study-level error analysis below). In this setting, the negative LOSO $R^{2}$ serves as a quantitative fingerprint of an information ceiling imposed by current reporting practices. This is directly aligned with recent discussions of why ML for polymeric long-acting injectables can plateau when formulation descriptors are incomplete or inconsistently defined across sources [5,19].

#### 3.8.1. Variance Decomposition by Study Identity

To quantify the fraction of total variance attributable to study-level effects, a one-way random-effects analysis (ICC-1) was performed separately for each target, using only studies that contributed at least two formulations with that target available [37,38]. For the release exponent *n*, 70.2% of the total variance was attributable to study identity (ICC = 0.702, p<0.001). For *K*, the between-study fraction was 63.8% (ICC = 0.638), and for Burst_24h, 45.9% (ICC = 0.459); all *F*-tests were significant at p<0.001. This subsetting yielded 51 studies (242 formulations) for Peppas *n* and *K* and 57 studies (265 formulations) for Burst_24h. These estimates show that a large fraction of the variance in fitted release parameters arises between laboratories rather than within formulations within a study. This is the variance component that LOSO exposes and that unreported process variables and in vitro protocol differences are expected to generate [3,9]. The lower ICC for Burst_24h is consistent with the idea that early time release is sensitive to within-study batch and surface heterogeneity [7,15].

#### 3.8.2. Study-Level Correlates of Cross-Study Error

To identify which study-level characteristics are associated with cross-study prediction error, per-study error (MAE under LOSO) was correlated with a panel of study-level reporting and heterogeneity proxies (number of formulations; fractions of formulations reporting $M_{w}$, unspecified molecular weight, and PDI; the number and entropy of distinct formulation methods; the number of distinct LA:GA ratios; and the median release-profile duration) using Spearman rank correlations. Most proxies showed no significant association with error, indicating that the coarse descriptors recoverable from the published record explain little of the between-study performance variation. Two associations reached nominal significance: median release-profile duration correlated positively with Burst_24h error (ρ=0.30, p=0.011, N=71 studies) and negatively with Peppas *K* error (ρ=−0.31, p=0.0012, N=105 studies), suggesting that studies characterizing longer release windows yield more stable rate-constant estimates while incurring greater burst-prediction error, consistent with the heightened sensitivity of early time release to unreported microstructural and processing detail. Given the number of proxies examined, these modest effects are best interpreted as exploratory rather than confirmatory; the more general finding is that the weakness of all available proxies, together with the high study-level variance components (Table 10), localizes interlaboratory variability to factors that are not captured by current reporting practice [3,6,9]. The differing study counts across these analyses (113 under LOSO, 33 in the per-study $R^{2}$ distribution, 51–57 in the ICC decomposition, and 71–105 in the error correlations) reflect their distinct inclusion rules: minimum formulation counts per study (≥3 for the distribution, ≥2 for the ICC, applied separately per target) and the availability of the relevant target and reporting proxies.

### 3.9. Uncertainty Quantification

**Figure 9:**
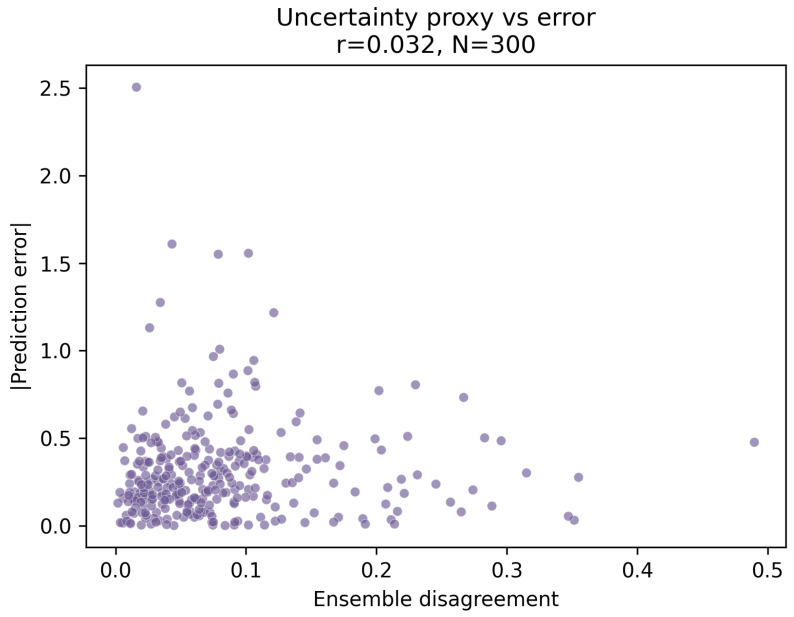
Uncertainty calibration for the release exponent *n*: ensemble disagreement versus absolute prediction error.

Prediction uncertainty was quantified using the standard deviation of base-learner predictions within each fold. For the release exponent *n*, ensemble disagreement showed little correlation with absolute prediction error in the current grouped-CV export (Pearson r=0.032, N=300; Figure 9), indicating that base-learner spread is not a calibrated reliability signal for this dataset. This weak relationship is expected when a substantial component of error likely reflects unmeasured study-level factors that are not recoverable from the reported feature set [39,40].

### 3.10. Reporting Gap Analysis

Table 11 cross-references variable availability with predictive importance and mechanistic relevance. Drug loading capacity and the hydrophilicity index were the two largest feature-importance contributors for Peppas *n* (14.9% and 12.7%, respectively), followed by drug encapsulation efficiency, particle size, and polymer molecular weight. Polymer molecular weight was reported in a standardized form ($M_{w}$) by 59% of formulations. Many studies instead reported an unspecified “molecular weight” value without distinguishing $M_{w}$ from $M_{n}$, and a small fraction reported only $M_{n}$. Because these reporting categories overlap across studies, availability is summarized at the formulation level. A standardized molecular weight ($M_{w}$ or $M_{n}$) was directly reported for 64% of formulations; for the remaining formulations, an unspecified numeric molecular weight was used where available, and any values still missing were resolved during dataset curation, so that the modeling feature Polymer MW was complete for all 321 formulations (Table 2). This distinction is important: the source literature contains missing or ambiguous fields, whereas the matrices used for modeling are complete; consequently, as shown in the leave-one-study-out analysis (Section 3.8), the choice of imputation method used inside the cross-validation folds does not affect the results. Critical polymer descriptors, including $M_{n}$ (5% of formulations) and polydispersity index (PDI, 4%), were too sparse to include despite their established roles in controlling chain-end density and molecular weight distribution breadth, which influence degradation kinetics and pore formation [1,3,4]. The harmonized formulation-method category (O/W, S/O/W, W/O/W, and related reported emulsion-route labels) was universally available but excluded from the primary numeric feature matrix to preserve a consistent numerical design space for leverage-based AD calculations. Exploratory models incorporating one-hot-encoded method indicators did not materially improve cross-validated performance ($\Delta R^{2}<0.01$), which suggests that method labels are insufficient surrogates for quantitative process descriptors [6,15].

Below these partially reported variables, Table 11 lists mechanistically important process-level variables that were not systematically or quantitatively extractable from the source literature: internal porosity, solvent removal rate, stirring or homogenization parameters, drying procedure, manufacturing scale, and polymer end-group chemistry. These factors govern early time release, skin formation, and the selection of the degradation pathway [3,7,15]. For end-group chemistry, some information may be recoverable from polymer grade names, but grade-level identifiers were inconsistently reported across studies, preventing systematic extraction at scale [1]. The asymmetry between variable importance and reporting completeness is notable: the most important single feature is frequently missing or ambiguously defined. In contrast, the process variables most likely to resolve the remaining variance are not quantitatively extractable. This pattern supports the interpretation that the predictive ceiling is set by field-wide reporting practices rather than by algorithmic limitations [5,19].

#### Proposed Minimum Reporting Elements

To improve cross-study reproducibility and enable predictive modeling that generalizes out of this study, the following minimum reporting standards are proposed:

- **Polymer characterization:** Both $M_{w}$ and $M_{n}$ (GPC), plus polydispersity index (PDI). Missing PDI and $M_{n}$ obscures the chain-length distribution that drives degradation heterogeneity [3,4].
- **End-group chemistry:** Explicit acid-terminated versus ester-capped status. This variable influences autocatalysis and degradation rate, and it is therefore expected to affect fitted rate constants such as *K* [1,3].
- **Solvent removal:** Evaporation temperature, duration, pressure conditions, and phase volumes. These parameters govern skin formation and internal porosity and are directly linked to burst magnitude [7,15].
- **Homogenization energy:** Stirring rate (rpm), duration, and impeller or probe type. These variables determine droplet size distributions and microstructure and are not captured by method labels alone [3,6].
- **Internal structure:** Quantitative internal porosity (e.g., via mercury porosimetry or micro-CT) and surface drug enrichment proxies, since these are primary drivers of burst and early time transport regimes [7,15].
- **Drying and process scale:** Post-formation drying procedure (e.g., lyophilization versus vacuum or air-drying) and manufacturing/batch scale, both of which influence solvent-removal kinetics, residual solvent, and the reproducibility of microstructure on scale-up [3,6].

Adoption of these elements would provide the process-level covariates needed to reduce inter-study variance and align PLGA formulation reporting with quality-by-design principles, which emphasize defining and controlling critical material attributes and process parameters [8]. These recommendations are also consistent with the broader direction of ML-enabled formulation design, where model utility depends on standardized, information-rich descriptors [5,18,19].

## 4. Limitations

Several limitations of this study should be acknowledged. First, the release data were extracted primarily from published figures using WebPlotDigitizer, introducing digitization noise on the order of a few percent of the ordinate value per point. This uncertainty propagates into the fitted Korsmeyer–Peppas parameters but is unlikely to explain the magnitude of the inter-study variance observed (the between-study variance components reported in Table 10 are far larger than plausible digitization noise). Second, protocol heterogeneity across source studies, including differences in dissolution medium (PBS versus SIF), sink conditions, agitation method, assay procedures, and sampling schedules, represents a confound that cannot be fully disentangled from the compositional and formulation variables modeled here; this between-laboratory heterogeneity is precisely what the negative leave-one-study-out $R^{2}$ and the high study-level ICC expose (Section 3.8, Table 10). Third, attributing the LOSO performance collapse to unreported process variables is a mechanistic interpretation supported by the reporting-gap analysis (Section 3.10) and the study-level error analysis (Section 3.8), but it is not a proven causal relationship. Unmeasured drug–polymer interactions, differences in polymer grade, and batch-to-batch PLGA variability may also contribute to between-study shifts. Fourth, burst release is highly sensitive to surface and internal microstructure, including surface porosity, skin thickness, and surface drug enrichment, which are rarely characterized in the source literature. This limits the interpretability and transportability of models for Burst_24h and helps explain the most negative leave-one-study-out value observed, that for burst ($R^{2}=-0.180$; Table 9). Finally, the compiled dataset is dominated by microparticles produced using emulsion-based methods, and the conclusions may not extend to spray-dried, electrosprayed, or microfluidic formulations, which are scarcely represented in the curated set, where emulsion routes account for the large majority of formulations (Table 1). These alternative techniques differ fundamentally in how droplets or particles are formed and solidified: spray drying atomizes the polymer–drug solution and removes solvent by rapid convective evaporation; electrospraying applies an electric field to generate fine, often narrowly distributed droplets; and microfluidic methods produce highly monodisperse droplets through controlled flow focusing. Each yields characteristic particle-size distributions, internal porosity, and surface morphologies, and may therefore exhibit burst and release behavior that differs systematically from the emulsion-based systems that dominate the present dataset.

## 5. Conclusions

**Figure 10:**
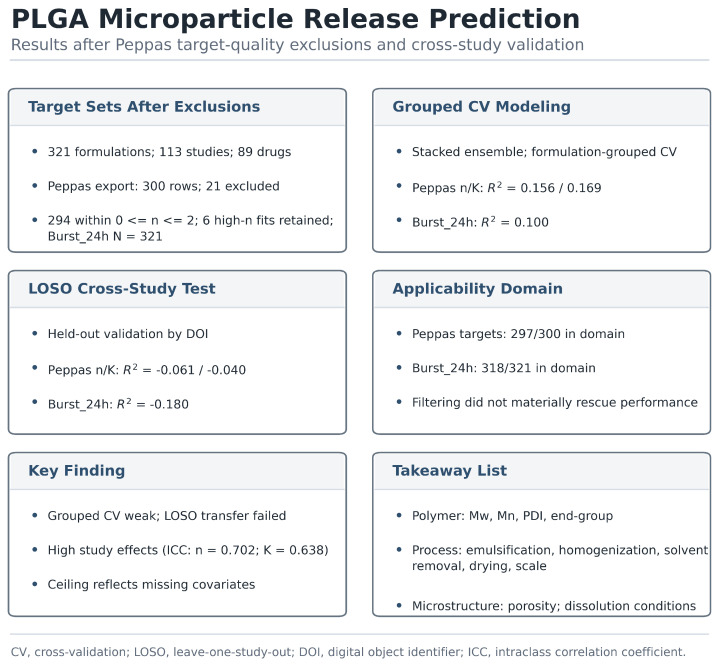
Results-focused graphical summary of this study. The two-partition boxes separate the analytical labels from the numerical findings after target-quality exclusions: the paired Peppas modeling export retained 300 formulations (294 within 0≤n≤2 and 6 retained out-of-window empirical fits), whereas Burst_24h analyses used all 321 formulations. Grouped cross-validation recovered only weak signal, leave-one-study-out validation failed to transfer across studies, and applicability-domain filtering did not rescue performance, motivating the listed reporting priorities.

Figure 10 provides a results-focused graphical summary of the target-accounting, model-performance, and reporting-priority findings.

This study evaluated the attainable predictability of poly(lactic-*co*-glycolic acid) (PLGA) microparticle release behavior when models are trained on covariates commonly reported across studies in the published literature. The analysis examined 321 formulations from 113 studies spanning 89 drugs, utilizing a previously curated literature-mined repository, and parameterized early time release using Korsmeyer–Peppas descriptors, with Peppas modeling performed on a 300-formulation subset. In grouped 10-fold cross-validation at the formulation level, the stacked ensemble achieved limited predictability for the release exponent *n* ($R^{2}=0.156$, MAE = 0.306, RMSE = 0.420). Predictability was similarly limited for the rate constant *K* ($R^{2}=0.169$) and for the 24 h burst ($R^{2}=0.100$), consistent with the heightened sensitivity of early time release to microstructure and processing conditions.

Benchmarking against linear regression, random forests, and gradient-boosted trees showed modest differences in performance and convergence of model families near the same information ceiling. Random forests performed best for the Peppas targets ($R^{2}=0.338$ for *n* and $R^{2}=0.276$ for *K*), but no model family produced high-fidelity prediction from the reported covariates alone. A robustness analysis further reinforced this interpretation. Augmenting the feature space with one-hot encoding of formulation method (e.g., O/W versus W/O/W) produced a negligible improvement in predictive performance ($\Delta R^{2}<0.01$), suggesting that coarse method labels do not substitute for quantitative descriptors of microstructure formation and processing energy input.

The most consequential finding emerged when generalization was tested across laboratories. Leave-one-study-out (LOSO) validation led to a collapse in performance for mechanistic targets, with pooled LOSO $R^{2}$ values of −0.061 for *n*, −0.040 for *K*, and −0.180 for Burst_24h. This indicates that models trained on literature covariates can perform worse than a mean predictor when applied to unseen studies. This behavior localizes the predictability ceiling to between-study heterogeneity and latent confounding rather than to within-study noise alone. Consistent with this conclusion, variance decomposition by study identity indicated that study-level effects account for a majority of the variance in fitted mechanistic parameters (ICC(1) = 0.702 for *n* and ICC(1) = 0.638 for *K*). At the same time, burst showed substantial but smaller study attribution (ICC(1) = 0.459). Together, LOSO failure and high ICC values provide quantitative evidence that between-study effects account for the variance component limiting cross-study modeling, consistent with a contribution from unreported or inconsistently reported process variables.

Burst behavior provided a complementary diagnostic of reporting limitations. Across the full set of 321 formulations, burst release was heavily skewed toward high early release under the binary 20% threshold, with 316 formulations (98.4%) in the high-burst class. Under this distribution, burst-risk classification collapsed to the majority class, yielding a macro-F1 of 0.496 despite high apparent accuracy. This highlights that the published record contains too few lower-burst examples and too little process metadata to support discriminative modeling of clinically desirable low-burst regimes. The limited burst regression performance ($R^{2}=0.100$) is therefore best interpreted as a signature of missing microstructure and processing covariates rather than as an intrinsic absence of structure in PLGA systems.

These results have direct implications for how machine learning should be used in formulation science. When applied to heterogeneous datasets derived from the published literature, model performance should be interpreted as a measurement of information content rather than as a definitive limit on the predictability of PLGA release. The present study shows that only a weak signal is recoverable within the reported covariate space under grouped validation, and that generalization across laboratories breaks down because the largest variance components lie outside that space. To move beyond the observed ceiling and enable reliable cross-study prediction, future PLGA microparticle reports should include standardized characterization of polymer molecular weight (including $M_{w}$ and $M_{n}$ with polydispersity), polymer end-group chemistry, quantitative solvent-removal protocols, explicit homogenization and emulsification parameters, and quantitative measures of internal porosity or microstructure. Adoption of these reporting elements would align the field more closely with quality-by-design principles and enable models to learn the process-to-microstructure-to-release relationships that are currently unidentifiable from published covariates.

In summary, the primary barrier to cross-study predictive modeling of PLGA microparticle release is not model capacity but the incompleteness and lack of standardization of the published feature space. Future progress in data-driven PLGA formulation design will therefore depend on improved reporting standards and on routine measurement and disclosure of the process variables that set microstructure and early time release behavior.

## 6. Reproducibility Details

All supervised analyses used one row per formulation. Grouped 10-fold cross-validation used Formulation Index as the grouping variable, and LOSO validation used DOI as the held-out study identifier. Within each outer validation split, missing-value imputation and feature scaling were fit on the training fold only and then applied to the held-out fold. The fixed model settings used for the primary analyses are summarized in Table 12; no grid search, Bayesian optimization, class reweighting, resampling, or synthetic augmentation was applied.

## Figures and Tables

**Table 1:** Composition of the curated dataset of 321 PLGA microparticle formulations: distribution of preparation methods, lactide:glycolide (LA:GA) ratios, polymer molecular-weight reporting, drug physicochemical classes, and the duration of the digitized release-profile window. Molecular-weight reporting categories are non-mutually exclusive (a study may report more than one field), so their percentages do not sum to 100%.

Attribute	Category	*N*	%
Preparation method	O/W	204	63.6
	S/O/W	74	23.1
	W/O/W	42	13.1
	S/W/O/W	1	0.3
LA:GA ratio	50:50	227	70.7
	75:25	85	26.5
	Other	9	2.8
Polymer MW reporting ^*a*^	$M_{w}$	190	59.2
	$M_{n}$	17	5.3
	Unspecified MW	127	39.6
	PDI	13	4.0
Drug molecular weight	<300 Da	68	21.2
	300–600 Da	213	66.4
	>600 Da	40	12.5
Drug lipophilicity (LogP)	Hydrophilic (<0)	38	11.8
	Moderate (0–3)	128	39.9
	Lipophilic (>3)	155	48.3
Release-profile window	≤7 h	13	4.0
	7–14 h	58	18.1
	14–28 h	83	25.9
	28–56 h	146	45.5
	56–84 h	7	2.2
	84–168 h	13	4.0
	168–365 h	1	0.3

^*a*^ Non-mutually exclusive reporting categories; a study may report multiple molecular-weight fields.

**Table 2:** Summary statistics of key inputs and targets across 321 formulations (formulation-level means shown). Polymer MW is in kDa; particle size is in μm; loading and encapsulation are in %. Peppas *n* is dimensionless; Peppas *n* and *K* are reported for the paired Peppas modeling export (N=300, including six high empirical fits with n>2). Peppas *K* was computed after expressing time in hours; because *K* depends on the fitted exponent *n*, values are descriptive fitted constants rather than directly comparable dimensional quantities across all formulations. Burst_24h is a unitless fraction. Values slightly above 1 can occur due to assay normalization and digitization noise [26].

Feature	Source	N	Mean ± SD	Min	Max
Drug MW	Drug property	321	432.41 ±232.60	130.08	1488.81
Drug LogP	Drug property	321	2.76 ±2.35	−11.55	9.11
Drug TPSA	Drug property	321	101.26 ±96.79	6.48	701.77
Polymer MW	Formulation	321	37.07 ±29.09	2.30	215.00
Particle Size	Formulation	321	46.04 ±46.46	1.15	295.70
Drug Loading Capacity	Formulation	321	11.79 ±9.42	0.02	61.90
Drug Encapsulation Efficiency	Formulation	321	67.07 ±22.88	1.31	100.00
Peppas *n*	Target	300	0.710 ±0.458	0.019	3.219
Peppas *K*	Target	300	0.209 ±0.203	0.000	1.357
Burst_24h	Target	321	0.811 ±0.197	0.032	1.082

**Table 3:** Target accounting for formulation-level modeling exports.

Dataset Step	Formulations
Total curated formulations	321
Valid Burst_24h targets used for burst regression/classification	321
Numerically estimated Peppas *K* values before paired-modeling restriction	321
Peppas paired modeling export	300
Peppas *K* rows retained in paired modeling export	300
Peppas *n* rows within 0≤n≤2 in paired modeling export	294
High empirical Peppas *n* rows (n>2) retained in paired modeling export	6
Formulations excluded from paired Peppas modeling export	21

**Table 4:** Stacked ensemble cross-validation performance (grouped 10-fold, identical protocol to benchmark comparison). Peppas targets are evaluated on the modeling subset (N=300); Burst_24h is evaluated on the full formulation set (N=321).

Target	$R^{2}$	MAE	RMSE
Peppas_n	0.156	0.306	0.420
Peppas_K	0.169	0.140	0.185
Burst_24h	0.100	0.144	0.186

**Table 5:** Benchmark comparison across model families under grouped 10-fold cross-validation (same evaluation units as Table 4).

Target	Model	$R^{2}$	MAE	RMSE
Peppas_n	Linear	0.090	0.302	0.422
Peppas_n	RandomForest	0.338	0.246	0.359
Peppas_n	XGBoost	0.300	0.245	0.370
Peppas_n	StackedEnsemble	0.156	0.306	0.420
Peppas_K	Linear	0.141	0.132	0.182
Peppas_K	RandomForest	0.276	0.114	0.167
Peppas_K	XGBoost	0.144	0.125	0.182
Peppas_K	StackedEnsemble	0.169	0.140	0.185
Burst_24h	Linear	0.011	0.150	0.195
Burst_24h	RandomForest	0.188	0.125	0.177
Burst_24h	XGBoost	0.090	0.130	0.187
Burst_24h	StackedEnsemble	0.100	0.144	0.186

**Table 6:** Features contributing >5% importance for prediction of release exponent *n* (random forest).

Feature	Importance (%)
Drug Loading Capacity	14.9
Hydrophilicity Index	12.7
Drug Encapsulation Efficiency	11.9
Particle Size	11.4
Polymer MW	8.7
Drug MW	6.3
ExactMolWt	5.9
Drug LogP	5.5

**Table 7:** Confusion matrix for two-class burst-risk classification (321 formulations). Accuracy = 0.984; macro-F1 = 0.496.

	Predicted		
Actual	Low/Mod	High	Recall
Low/Mod (<20%)	0	5	0.00
High (≥20%)	0	316	1.00
Precision	0.00	0.984	

**Table 8:** Applicability-domain analysis: formulation-level leverage with $h^{*}=3p/N$ computed per target.

Target	Region	Count	$R^{2}$	MAE	$h^{*}$
Peppas_n	In-domain	297	0.160	0.305	0.150
	High-leverage	3	–	–	
Peppas_K	In-domain	297	0.174	0.138	0.150
	High-leverage	3	–	–	
Burst_24h	In-domain	318	0.101	0.144	0.140
	High-leverage	3	–	–	

**Table 9:** Comparison of grouped 10-fold CV and leave-one-study-out (LOSO) validation (formulation-level evaluation).

	Grouped 10-Fold CV			LOSO		
Target	$R^{2}$	MAE	RMSE	$R^{2}$	MAE	RMSE
Peppas_n	0.156	0.306	0.420	−0.061	0.363	0.498
Peppas_K	0.169	0.140	0.185	−0.040	0.142	0.179
Burst_24h	0.100	0.144	0.186	−0.180	0.202	0.245

**Table 10:** Variance decomposition of release parameters by study identity (ICC-1, one-way random effects model). Each target uses only studies contributing ≥2 formulations with that target available.

Target	Studies	Formulations	ICC(1)	*F*-Test *p*
Peppas_n	51	242	**0.702 **	<0.001
Peppas_K	51	242	**0.638**	<0.001
Burst_24h	57	265	**0.459**	<0.001

**Table 11:** Reporting gap analysis: variable availability versus predictive importance and mechanistic relevance. Variables below the mid-rule were not systematically or quantitatively extractable from the source literature but are established mechanistic drivers of release behavior.

Variable	Category	Extractable (%; Level)	Importance (%)	Mechanistic Role	In Model
Drug loading	Formulation	100	14.9	Initial payload, drug–polymer interaction	Yes
Hydrophilicity index	Composite	64 ^*d*^	12.7	Polymer aqueous accessibility proxy	Yes
Encapsulation eff.	Formulation	100	11.9	Process efficiency proxy	Yes
Particle size	Formulation	100	11.4	Surface area, diffusion path	Yes
Polymer MW ^*a*^	Polymer	64 ^*c*^	8.7	Degradation rate, chain mobility	Yes (composite)
Polymer $M_{w}$	Polymer	59	–	Weight-average MW; preferred source	Yes ^*b*^
Polymer $M_{n}$	Polymer	5	–	Number-average MW; chain-end density	No (94.7% missing)
PDI	Polymer	4	–	MW distribution breadth	No (96% missing)
Formulation method	Process	100	–	Microstructure determinant	No (categorical)
Drug MW	Drug	100	6.3	Diffusivity, solubility	Yes (RDKit)
ExactMolWt	Drug	100	5.9	Molecular size descriptor	Yes (RDKit)
Drug LogP	Drug	100	5.5	Lipophilicity, partitioning	Yes (RDKit)
Internal porosity	Microstructure	0	–	Pore-mediated diffusion	No (not systematically extractable)
Solvent removal rate	Process	0	–	Skin formation, porosity	No (not systematically extractable)
Stirring/homogenization	Process	0	–	Droplet size, uniformity	No (not systematically extractable)
End-group chemistry	Polymer	0	–	End-group effects on degradation	No (not systematically extractable)
Drying procedure	Process	0	–	Residual solvent, skin/microstructure	No (not systematically extractable)
Manufacturing scale	Process	0	–	Scale-dependent mixing and solvent removal	No (not systematically extractable)

^*a*^ Composite of $M_{w}$, $M_{n}$, or unspecified MW (priority: $M_{w}>M_{n}>$ unspecified). ^*b*^ Contributes to composite Polymer MW when available. ^*c*^ Formulation-level availability after harmonization; study-level reporting categories are non-mutually exclusive. ^*d*^ Computed composite feature; extractability limited by availability of Polymer MW.

**Table 12:** Reproducibility settings for the primary modeling workflow.

Component	Setting
**Feature Matrix**	Fixed numeric 15-feature formulation-level matrix; one row per formulation index.
**Preprocessing**	Mean imputation and standard scaling were fit within each training fold only.
**Random forest base learner**	200 trees; maximum depth = 10; random seed = 42.
**XGBoost base learner**	200 trees; maximum depth = 6; learning rate = 0.05; squared-error regression objective.
**RBF-SVR base learner**	Penalty parameter C=10; kernel coefficient set to scale.
**Stacked meta-learner**	Ridge regression with α=1.0; internal stacking cross-validation used five folds.
**Benchmark models**	Linear regression, random forest, and XGBoost regressor were fit as comparator models using the same fold assignments. Random forest used 100 trees, maximum depth = 10, and random seed = 42; XGBoost used 100 trees, maximum depth = 6, and learning rate = 0.05.
**Burst classifier**	XGBoost binary classifier with 200 trees, maximum depth = 6, and learning rate = 0.05; binary threshold for Burst_24h was 0.20.
**Validation**	Grouped 10-fold cross-validation by formulation index; LOSO validation by DOI; burst holdout split 80/20 by formulation as an additional leakage check.
**Software environment**	Python 3.13.5; numpy 2.3.5; pandas 2.3.2; scikit-learn 1.7.1; xgboost 3.1.3; RDKit 2025.9.4; scipy 1.16.1; statsmodels 0.14.6; openpyxl 3.1.5.
**Code version**	Public analysis repository listed in data availability.

## Data Availability

The raw data used in this study were obtained from Mendeley Data [42]. Supplementary Table S1 accompanies this submission and lists the source DOI inventory. The Python code and reproducibility workflow used for the analysis are available at https://github.com/aryanpshah/plga-microparticle-release-ml (accessed on 21 June 2026).

## Supplementary Materials

The following supporting information can be downloaded at: https://www.mdpi.com/article/10.3390/pharmaceutics18070805/s1, Supplementary Table S1 (Source DOI inventory); Supplementary Table S2 (Modeling outputs); Supplementary Table S3 (Harmonized formulation method categories and frequencies); and Supplementary Dataset S1 (Dataset PLGA microparticles).
